# The nucleus as a mechanobiological hub in muscle aging

**DOI:** 10.1080/19491034.2026.2688584

**Published:** 2026-06-18

**Authors:** Osman Esen, Edmund Battey, Matthew J. Stroud, Tyler J. Kirby

**Affiliations:** a Department of Physiology, Amsterdam University Medical Center, Vrije Universiteit Amsterdam, Amsterdam, The Netherlands; b Department of Biomedical Sciences, Faculty of Medical and Health Sciences, University of Copenhagen, Copenhagen, Denmark; c British Heart Foundation Centre of Research Excellence, School of Cardiovascular Medicine and Sciences, King’s College London, London, UK; d Heart Failure & Arrhythmias, Amsterdam Cardiovascular Sciences, Amsterdam, The Netherlands; e Tissue Function & Regeneration, Amsterdam Movement Sciences, Amsterdam, The Netherlands; f Division of Cardiovascular Medicine, Department of Internal Medicine, University of Kentucky, Lexington, KY, USA

**Keywords:** Skeletal muscle, aging, extracellular matrix, cytoskeleton, LINC complex, nuclear lamina, nucleus, mechanotransduction

## Abstract

Aging leads to a progressive loss of muscle mass and strength, termed sarcopenia, which is accelerated by inactivity and exacerbated by intrinsic cellular and molecular dysfunctions within the muscle fiber. Central to these changes is mechanotransduction, the process by which mechanical stimuli are converted into biochemical cues critical for protein synthesis, cytoskeletal remodeling, calcium signaling, and metabolism. Recent evidence highlights the nucleus as a key mechanosensory organelle in skeletal muscle. Forces transmitted from the extracellular matrix (ECM) through the cytoskeleton reach the nuclear envelope, where the Linker of Nucleoskeleton and Cytoskeleton (LINC) complex and nuclear lamina convert physical stress into gene-regulatory events. Aging may alter these structures, producing changes in nuclear morphology, decreased stiffness, envelope fragility, and compromised transcriptional control. This review examines how the ECM, cytoskeleton, LINC complex, and nuclear lamina change in aged skeletal muscle, proposing that impaired nuclear mechanosignaling contributes to muscle fiber dysfunction during physiological aging.

## Introduction

Aging is a complex, multifactorial phenomenon that significantly impacts skeletal muscle health. One of the hallmarks of aging is the progressive loss of muscle mass and strength, known as sarcopenia [1,2]. While reductions in physical activity contribute to this condition, emerging evidence suggests that sarcopenia is driven by intrinsic cellular and molecular alterations that compromise skeletal muscle homeostasis [2–4]. Central among these mechanisms is mechanotransduction, the process by which mechanical stimuli are converted into biochemical signals that regulate muscle maintenance and adaptation [5,6]. Mechanotransduction is critical for muscle health, as its disruption is a shared feature in both disuse atrophy (from mechanical unloading) and muscular dystrophy (from genetic mutations, typically causing progressive, irreversible damage) [7,8]. Mechanotransduction pathways regulate muscle protein synthesis [9,10], calcium signaling [11–13], cytoskeletal remodeling [14,15], stem cell activity [16], and energy metabolism [17].

**Figure 1.**
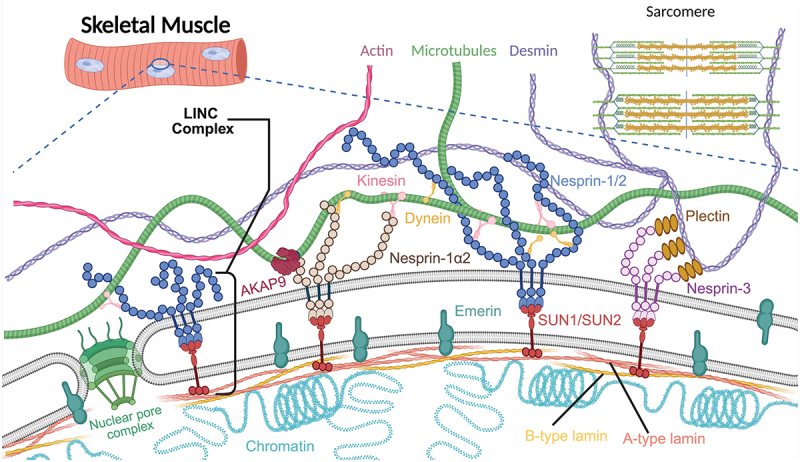
Schematic illustration of the nuclear mechanotransductive pathway in skeletal muscle fibers. The cytoskeleton proteins (desmin, microtubules, and F-actin) are physically connected to the myonuclei via the Linker of Nucleoskeleton and cytoskeleton (LINC) complex composed of nesprin and SUN protein isoforms. Cytoskeleton elements are connected to the nesprin proteins located on the outer nuclear membrane, which are connected in the nuclear membrane space with the SUN domain proteins located at the inner nuclear membrane. These SUN domain proteins are interacting with the nuclear lamina (composed of A- and B-type lamins), creating the physical link with chromatin.

In recent years, the cell nucleus has emerged as a key mechanotransductive hub [18–20], with nuclear alterations linked to mechanosensitivity loss in aging tissues [21–24]. Changes in nuclear morphology are typically attributed to either changes in cytoskeletal forces acting on the nucleus [25,26], or changes to structures within the nucleus determine its mechanical properties (i.e. nuclear lamina and chromatin) [27–30], or a combination of both. To this end, multiple studies have reported changes in nuclear morphology in aged skeletal muscle fibers of both rodents and humans [3,31–33], while we have reported that the morphology of aged myonuclei is influenced by physical activity [3], suggesting that perhaps there is altered nuclear mechanotransduction in aged muscle fibers [34]. Central to nuclear mechanotransduction are the Linker of Nucleoskeleton and Cytoskeleton (LINC) complex and nuclear lamina [35,36]. The LINC complex spans the nuclear envelope, linking the cytoskeleton to the nucleoskeleton and thereby acting as an indirect mechanical connection point between the extracellular matrix (ECM) and the genome [37–40]. Meanwhile, the nuclear lamina, composed primarily of A- and B-type lamins, provides structural integrity to the nucleus [29], modulates chromatin organization, and gene expression in response to mechanical stress [41–43]. Although nuclear mechanotransduction was initially viewed primarily as an ‘outside-in’ process (transmitting external forces to the nucleus), a growing body of literature now demonstrates its bidirectional nature, with nuclear architecture and composition influencing cytoskeletal organization and cytoplasmic responses (‘inside-out’) [19,44]. Figure 1 illustrates the key components involved in the nuclear mechanotransduction pathway in skeletal muscle fibers.

Given their central roles in nuclear organization and force transduction in both ‘inside-out’ and ‘outside-in,’ the LINC complex and nuclear lamina represent critical nodes in the mechanobiological network in skeletal muscle [22]. This review summarizes the primary structures and networks involved in nuclear mechanotransduction in skeletal muscle, what is known about how aging may affect these structures and networks, and highlights directions for future research on nuclear mechanobiology during skeletal muscle aging. Importantly, we will highlight the unique cytoskeletal and multinuclear organization of skeletal muscle fibers, which likely results in distinctive nuclear mechanosignaling mechanisms compared to mononucleated cells or cells in culture, although whether this is the case remains to be tested.

## Extracellular matrix

**Figure 2.**
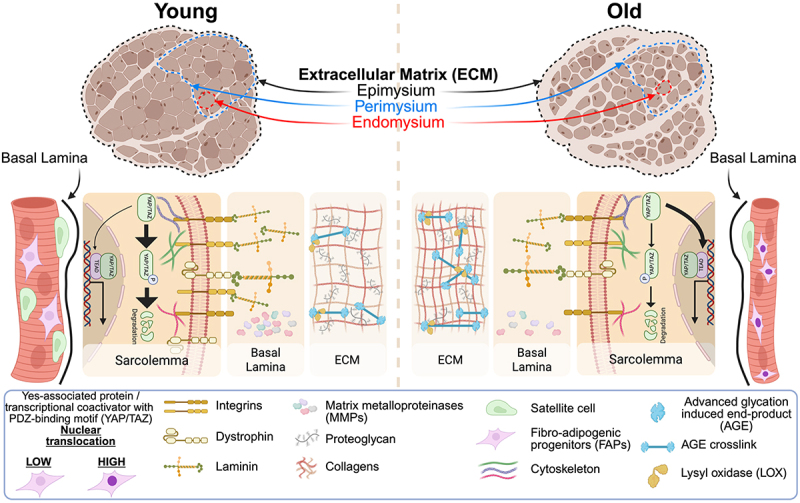
Schematic illustration of the age-dependent extracellular matrix (ECM) remodeling in skeletal muscle tissue. The age-dependent ECM remodeling induces fibrosis characterized by a denser ECM with increased collagen crosslinking density, accumulation of advanced glycation end-products (AGEs), decreased laminin and focal adhesion points, and decreased matrix metalloproteinase expression. These changes lead to a decline in muscle stem cell activity and increased YAP/TAZ nuclear translocation in FAPs and satellite cells.

The muscle ECM is structured into three distinct layers: the endomysium, perimysium, and epimysium, which surround individual muscle fibers, fiber bundles, and whole muscles, respectively [45,46] (Figure 2). Composed primarily of fibrillar collagens (type I and III), collagen IV, laminins, glycoproteins, and proteoglycans, the ECM forms a complex and highly organized network important for muscle fiber force transmission [45,47], maintenance [45], ensures structural stability [45,47], regulates biochemical signaling [45,47] and repair [45,47,48]. The cell–matrix interface functions as the starting point of the ‘outside-in’ mechanotransduction, where integrins connect the cytoskeleton to the ECM via adhesion proteins [47]. They serve as both attachment sites and mechanosensors [49,50]. The sensed mechanical cues are transmitted through the cytoskeleton, eventually reaching the nucleus through the LINC complex [18,51]. This provides a continuous mechanical pathway from the sarcomere and ECM to the nucleus [51], thereby enabling force-sensitive modulation of nuclear architecture and gene expression [18,23,52]. In healthy muscle, each myonucleus is located in very close proximity to the cell membrane and thus, the ECM. Such spatial proximity likely facilitates efficient force transmission between the ECM and the nucleus. In addition, many stem cell populations that are critical for muscle regeneration and implicated in muscle aging reside within the extracellular niche [53].

### Extracellular matrix composition and organizational changes with aging

With aging, skeletal muscle ECM undergoes profound alterations that significantly contribute to reduced muscle quality and regenerative capacity [47,54,55]. A hallmark of aged muscle is fibrosis [48,54], characterized by increased accumulation and cross-linking of collagen fibers [48,56–58], which has been reported to stiffen the ECM [59] up to 35-fold in the tibialis anterior and soleus muscles of old mice (24–30 months) compared to young (4-month) mice [60,61] (Table 1). Beyond ECM stiffening, age-dependent alterations in ECM organization have been reported [47,54]. In particular, reduced collagen tortuosity in muscles such as the gastrocnemius, flexor digitorum profundus, and soleus muscle of 22–24-month-old versus 3–4-month-old rodents. This decline in collagen tortuosity with aging renders the collagen network less wavy and more taut [54]. However, the overall alignment of most collagen fibers remains perpendicular to the muscle fibers regardless of the rodents age [47,54]. Such architectural changes reduce the ECM’s ability to deform in response to tensile forces generated during muscle contraction, rendering the muscle stiffer, especially along the circumferential axis of fiber alignment [54,62]. Techniques like second harmonic generation imaging and biaxial mechanical testing confirm that aging induces mechanical anisotropy (direction-dependent stiffness) of muscle due to altered collagen structure in rodents [54,62,63]. Collectively, these age-related changes in ECM composition and organization may alter the mechanoresponsiveness of the muscle fiber in rodents. In humans, the passive stiffness of muscle bundles was higher in older subjects compared to younger subject (mean age of 67 vs 21, respectively), whereas passive stiffness within individual fibers was unchanged, indicating the increase in stiffness may arise from the ECM [64]. Indeed, there was a significant accumulation of ECM in the aged individuals, rather than a change in the mechanical properties [64].

**Table 1.** Summary of the age-related changes to nuclear mechanotransductive elements in skeletal muscle. All changes are shown and old compared to young.

Mechanotransduction Element	Species	Cell or tissue source	Age comparison	Age-related changes	Study
Extracellular matrix	Mouse	MuSCs	2 mo & 14 mo	MuSC proliferation ↓ TIMP activity ↓ Focal adhesion points ↓ YAP/TAZ signaling ↑	Haroon et al. [16]
		Gastrocnemius	3–5 mo & 20–24 mo	Muscle ECM stiffness ↑ MMP activity ↓ TIMP activity ↓	Chen et al. [69]
		Extensor digitorum longus, FAPs and MuSCs	2 mo & 18 mo	Muscle ECM stiffness ↑ MMP activity ↓	Bobadilla Muñoz et al. [70]
		Diaphragm, extensor digitorum longus	3 mo & 6 mo & 12 mo & 18–22 mo	Laminin ↓	Lee et al. [75]
		Gastrocnemius & fibroblasts	3–4 mo & 22–24 mo	Muscle ECM stiffness ↑	Stearns-Reider et al. [54]
		Tibialis anterior	8–12 mo & 28–30 mo	Muscle ECM stiffness ↑ Muscle fiber stiffness =	Wood et al. [59]
	Rat	Tibialis anterior	4 mo & 28–30 mo	Muscle ECM stiffness ↑	Gao et al. [60]
		Soleus	4 mo & 24 mo	Muscle ECM stiffness ↑	Rosant et al. [61]
		Gastrocnemius, flexor digitorum profundus, soleus and blood	3 mo & 21 mo	Muscle ECM stiffness ↑ MMP activity ↓	de Sousa Neto et al. [65]
	Rats	Extensor digitorum longus, soleus	5–6 mo & 21–25 mo	Central nucleation ↑ ECM accumulation ↑	Ansved & Edström [136]
	Human	MuSCs	unknown	YAP/TAZ signaling ↑	Stearns-Reider et al. [54]
	Human	Vastus lateralis	25 +/- 3 yr & 78 +/- 6 yr	Collagen content = Collagen crosslinking = AGEs ↑	Haus et al. [56]
	Human	Vastus lateralis	~21 yr & 67 yr	ECM deposition ↑ Passive stiffness of muscle bundles ↑	Paven et al. [64]
Cytoskeleton	Mouse	Extensor digitorum longus, soleus	2 mo & 14 mo & 23 mo	Myonuclear Domain ↑ MT lattice ↓ Perinuclear MT ↑	Bruusgaard et al. [31]
	Rats	Plantar flexor muscles	6–8 mo & 24 mo	Desmin Expression ↑ Force production ↓ Muscle mass =	Russ & Grandy [133]
		Extensor digitorum longus, soleus	5–6 mo & 21–25 mo	Desmin ↑	Ansved &Edström [136]
Nuclear lamina	Mouse	Quadriceps, flexor digitorum brevis	2–5 mo & 24–28 mo	Nuclear invagination ↑ YAP/TAZ signaling ↑ Lamin A/C = Lamin B1 ↓	Iyer et al. [83]
		Gastrocnemius	1 mo & 3 mo & 6 mo	Lamin A/C ↓	Xiong et al. [181]
		Tibialis anterior, Extensor digitorum longus, soleus, gastrocnemius, diaphragm	3 mo & 12 mo & 24 mo	Lamin A/C ↓ DNA Damage ↑	Gao et al. [202]
		Tibialis anterior, Extensor digitorum longus, soleus, gastrocnemius, quadriceps	3–5 mo & 22 mo	Lamin B1 ↓ DNA Damage ↑	Zhang et al. [208]
	Human	Vastus lateralis	16–71 years	Lamin A transcript ↑ Lamin A =	Luo et al. [178]

### Mechanisms of ECM remodeling during aging

Aged muscle ECM is associated with a decline in the activity of matrix metalloproteinases (MMPs), which degrade ECM [48,63,65] and increased collagen crosslinking through both enzymatic mechanisms, principally lysyl oxidase (LOX)-mediated oxidative deamination, and non-enzymatic glycation via advanced glycation end-products (AGEs) [56,66–68]. The reduced degradation capacity leads to a net accumulation of ECM components, further exacerbating fibrosis [48,63]. Studies in aged rats (>21 months) and mice (>20 months) showed decreased levels of *MMP-2* [65,69], *MMP-3* [69], *MMP-9* [65], and *MMP-10* [70], indicating reduced systemic ECM remodeling capacity. However, resistance training in old rats increased *MMP-2* activity in skeletal muscle [65], suggesting that the age-dependent decline in MMP activity can be partially reversed with exercise.

Aging may also disrupt the fine balance between MMPs and their inhibitors, Tissue Inhibitors of Metalloproteinases (TIMPs) [65,69,71]. Additionally, studies have demonstrated a decrease in laminin concentration in the basal lamina of aging skeletal muscle, particularly in slow-twitch muscles [72,73]. This reduction in laminin concentration likely affects integrin-mediated adhesion, proper force transmission, and adaptation [47,72,74]. Functional studies also report that the loss of laminin α4 at the neuromuscular junction (NMJ) with aging is associated with impaired neuromuscular transmission and morphology [75], highlighting laminin’s importance in muscle physiology, which declines with age [75,76]. Finally, nuclear envelope proteins may have both indirect and direct roles in the modulation of profibrotic signaling, which has been shown in the context of cardiac hypertrophy [77]. Nevertheless, limited evidence exists showing direct effects of age-related changes in ECM structure or stiffness on mechanotransductive events within the muscle fiber, warranting further investigation.

### Muscle microenvironment and the stem cell niche

Much of the work looking at the effect of skeletal muscle aging and ECM structure on mechanotransduction has focused on the various muscle stem cell populations that interact with the ECM. Dysregulated ECM composition may influence the mechanotransduction of progenitor cells, such as satellite cells (SCs) and fibro/adipogenic progenitors (FAPs), residing in the skeletal muscle microenvironment, which could compromise skeletal muscle regenerative potential [16,48,78]. An important downstream consequence of increased ECM stiffness is enhanced mechanosensing via Yes-associated protein/transcriptional coactivator with PDZ-binding motif (YAP/TAZ) nuclear translocation [79–81], core components of the Hippo signaling pathway. Hyperactivation of Hippo signaling in SCs and FAPs promotes pro-fibrotic and anti-myogenic signaling, which may exacerbate fibrosis and impair regeneration [54,63,81]. During aging, the increased ECM stiffness may enhance YAP/TAZ activation, especially in areas undergoing active fibrosis [54]. Increased ECM stiffness is associated with increased YAP/TAZ activation in fibroblasts, which promotes fibrogenic conversion of SCs at the expense of their myogenic potential [54]. In addition, YAP/TAZ works cooperatively with the canonical pro-fibrotic TGFβ signaling pathway to promote the conversion of FAPs into myofibroblasts, which can be prevented by inhibiting the mechanosensitivity of FAPs [82]. These findings suggest that age-related muscle stiffness directly drives YAP/TAZ activation in FAPs, leading to ECM changes that could shift SC fate toward fibrosis [54,82]. YAP/TAZ activation in aged muscle has been observed not only in SCs and FAPs, but also in whole muscle lysates of aged animals (age 24–28 months) when compared to younger animals (age 2–5 months) [83]. Since lysates are composed of a mix of SCs, FAPs, and muscle fibers, it’s possible that aged myonuclei are also contributing to the increased YAP activation. Whether ECM stiffness directly drives YAP/TAZ activation in myonuclei has not been established in aged muscle fibers. More refined experimental approaches, such as immunofluorescence of YAP/ TAZ in muscle fibers, enzymatic digestion to isolate single fibers, or leveraging single-nucleus sequencing studies [84], could help clarify which cell types primarily contribute to the Hippo pathway activation.

Collectively, age-related changes in skeletal muscle ECM, particularly increased stiffness, altered collagen organization, and reduced matrix turnover, disrupt the physical scaffold around muscle tissue and the mechanical cues transmitted to the intracellular environment. The ECM provides structural support to the tissue and transmits mechanical cues to the cell, in part mediated by nuclear mechanosensing [85]. If we again focus on the well-characterized mechanosensitive YAP, nuclear wrinkling is modulated by the stiffness of the ECM, and correlates inversely with YAP nuclear localization in cultured cancer cells [86]. YAP nuclear localization can be directly modulated by tension on nuclear pores, which promotes nuclear entry in cell culture models [87]. In addition, changes in cytoskeletal organization and the connections between the cytoskeleton and the nucleus can change nucleocytoplasmic transport rates by modulating guanosine triphosphate (GTP) availability [88]. Finally, it was recently shown that aged MuSCs exhibited significantly lower growth rate and reduced integrin-α7 expression, with YAP nuclear localization significantly higher than in young SCs, while, paradoxically, YAP target genes *Cyr61* and *Ctgf* were substantially downregulated [16]. Thus, during aging, increased rigidity and altered compliance of the ECM may impose abnormal mechanical loads on the cytoskeleton, which could then transmit these forces to the nucleus. As such, the ECM may act as a critical upstream driver of cytoskeletal remodeling and nuclear mechanobiological dysfunction in aged muscle (Figure 2). Nevertheless, while the link between changes to the ECM and nuclear mechanotransduction in muscle stem cells is easy to envision, whether similar mechanisms exist in the muscle fiber, and whether this occurs through integrin-mediated mechanisms [74] or alternative mechanisms, remains an open area of investigation.

## Cytoskeleton

The cytoskeleton in skeletal muscle is highly organized and can broadly be divided into two main groups, namely the sarcomeric and non-sarcomeric cytoskeleton [89,90]. Each group contains distinct sets of proteins suited to its structural and functional roles. The sarcomeric cytoskeleton is composed of actin, myosin (myosin heavy chain II or sarcomeric myosin), titin, nebulin, α-actinin, myomesin, and troponin, among others, all proteins required for assembling the repeating sarcomere units and orchestrating muscle contraction for force generation [89–91]. The non-sarcomeric cytoskeleton, a dynamic network of filamentous proteins, is composed of three main types: actin filaments, microtubules (MTs), and intermediate filaments, with desmin being the most abundant intermediate filament in skeletal muscle [21,22,92,93]. This non-sarcomeric cytoskeleton protein network collectively maintains cellular and nuclear integrity [92,94], mediates intracellular transport [93], and transduces mechanical signals from the ECM to the nucleus [22]. In skeletal muscle, the non-sarcomeric cytoskeleton is highly specialized to accommodate the high mechanical demands placed on muscle fibers during contraction, stretch, and load-bearing [22,89]. To cope with such high mechanical demands, each non-sarcomeric cytoskeletal element plays a unique yet cooperative role within this structural framework [95–97]. A prominent example supporting the critical role of the non-sarcomeric cytoskeleton in skeletal muscle is seen in desmin-related myopathies, characterized by the absence or aggregation of desmin [98,99]. In these muscle fibers, mechanical force leads to disorganization of myofibrils, Z-line streaming, and the cytoskeletal collapse, leading to muscle damage and greater muscle weakness compared to normal muscle [98,99]. The role of the non-sarcomeric cytoskeleton on nuclear mechanics in striated muscle has emerged in recent years, with much of the seminal work being performed in cardiac myocytes [100–102].

### Actin filaments

The actin cytoskeleton is composed of globular actin (G-actin) subunits that polymerize to create long and flexible filamentous actin (F-actin) [103–105]. In mammals, six actin isoforms can be grouped into four muscle-specific actin isoforms: (skeletal muscle α-actin, smooth muscle α-actin (vascular), cardiac muscle α-actin, smooth muscle γ-actin (enteric)); and two non-muscle actin isoforms: (β-actin, γ-actin) [106]. In mononucleated cells, these filaments assemble into extensive networks throughout the cytoplasm and perinuclear region, where they connect to the nuclear envelope via the LINC complex [107]. When extracellular forces, such as changes in substrate stiffness, shear stress, or mechanical strain, are applied to the cell, these forces are transmitted from sites of cell-matrix adhesion through the actin cytoskeleton and the LINC complex, directly to the nucleus [107–109]. This mechanotransductive pathway enables cultured cells to perceive and react to mechanical cues by alternating global chromatin organization and local chromatin stretch, resulting in transcriptional activation [42,52]. In addition, actin dynamics have been shown to regulate the nucleo-cytoplasmic shuttling of mechanosensitive transcription factors [110]. However, this actin-mediated pathway is likely different in skeletal muscle fibers because of their unique specialization and architecture, with most skeletal muscle α-actin existing within the actin-myosin-based sarcomeres [89,111]. While there is some evidence that a portion of the γ-actin population tethers at costameres and colocalizes with dystrophin [112], the role of non-sarcomeric actin in skeletal muscle mechanotransduction is still unclear. Thus, for this review, we will focus on the other two classes of non-sarcomeric cytoskeletal elements, MTs, and intermediate filaments (i.e. desmin) [113], as we propose they are more likely to be the main contributors to nuclear mechanotransduction in skeletal muscle.

### Microtubules

MTs are highly dynamic, filamentous proteins composed of α- and β-subunits. In combination with the actin network, they are critical for myoblast fusion during muscle development, peripheral positioning of myonuclei in mature muscle fibers, and proper assembly and maintenance of the sarcomere [21,49,114,115]. MTs also act as intracellular transport highways, facilitating the positioning and movement of organelles, vesicles, RNA, and signaling molecules necessary for muscle fiber function and homeostasis [21,93,116]. During myogenesis, MTs provide a framework to guide myotube formation and maturation toward mature contracting muscle fibers, with mature muscle fibers displaying a mesh-like MT network. These mesh-like networks, known as MT lattices, are mainly located in the cortex of muscle fibers, forming a supportive cage that preserves the internal structure of the muscle fiber [21,117,118] (Figure 1). Maintaining this stable MT lattice structure is achieved through a fine balance in the post-translational modifications (PTMs) [15], such as acetylation, tyrosination, and phosphorylation [119]. Together, these PTMs finely tune the balance between MT stability and dynamics, regulating the rate of polymerization and depolymerization required for MT remodeling during cell division, differentiation, and motility [49,120]. Acetylation of α-tubulin, particularly at amino acid lysine 40, has been modeled to increase MT flexibility and resistance to mechanical stress based on cryo-EM structural studies [121], although many questions about the precise function of MT acetylation remain [122]. Nevertheless, increasing MT acetylation in skeletal muscle fibers using genetic and pharmacologic strategies increases cytoskeletal viscoelastic resistance [14]. Tyrosinated α-tubulin marks dynamic, growing MTs, while detyrosinated α-tubulin signifies stable, long-lived MTs [123,124]. In cardiomyocytes, detyrosination suppresses dynamic instability, prolonging Z-disk residency and enabling desmin to bind and reinforce the MT plus-end, leading to increased cytoskeletal stiffness and enhanced mechanotransduction [123,125,126]. This stabilized network resists contraction-induced deformation and supports efficient force transmission. In addition to acetylation and detyrosination, other modifications are associated with stabilized MTs in muscle cells, including the dephosphorylation of microtubule-associated protein 4 (MAP4), an MT binding protein, which stabilizes the MT for densification [123].

For example, in the *mdx* mouse model, which is widely used as a model for Duchenne muscular dystrophy (where dystrophin expression has been ablated), isolated muscle fibers had elevated levels of MT acetylation and detyrosination, accompanied by a decline in isometric force production due to increased cytoskeletal stiffness [15]. Similar measurements correlating MT remodeling with isometric force measurements remain absent. However, it is thought that elevated cytoskeletal stiffness can push muscle fibers outside a ‘goldilocks zone’ of optimal stiffness, causing either hypersensitivity or insensitivity in certain mechanotransduction pathways [49]. For example, this disruption may interfere with calcium signaling and reactive oxygen species (ROS) production essential for muscle function and adaptation, as has been observed in *mdx* mice [127–129]. Aside from a change in mechanical load, chronic oxidative stress and pro-inflammatory signaling with aging can induce MT rearrangements, and increased cytoskeletal stiffness impairs the ability of MTs to properly sense mechanical load [49,129,130]. Nevertheless, whether similar mechanisms are occurring in aged muscle fibers remains to be established. We propose that alterations in MT stiffness due to disrupted MT organization may lead to dysregulation in mechanotransductive pathways in aged muscle fibers.

### Desmin

Desmin is a type-III muscle-specific intermediate filament protein that polymerizes to form a stable cytoplasmic filamentous network, forming a robust scaffold linking the Z-discs of the sarcomere to one another and the plasma membrane [95–97] (Figure 1). This filament network ensures structural alignment to maintain the structural integrity and mechanical resilience, and proper force transmission during muscle contraction [95–97,131]. Desmin filaments stabilize the alignment of myofibrils to permit effective force transmission across the muscle fiber and connect the sarcomeres with cellular organelles (i.e. nucleus and mitochondria) and the sarcolemma [95–97]. It also plays a role in the adaptive response of muscle to mechanical stress and remodeling. Mutations in desmin lead to desminopathies [98], characterized by desmin aggregates that disrupt fiber architecture, leading to progressive muscle weakness and increased susceptibility to mechanical stress [132].

Studies have shown an increase in desmin expression in aging muscle [133,134]. Despite increased desmin levels, aged muscle often shows compromised function, possibly due to altered desmin organization, aggregation, or abnormal PTMs that could affect the transmission and dissipation of contractile force [133,134]. Desmin misalignment, in particular, disrupts the lateral transmission of force, and altered desmin expression and organization have been observed in aged and diseased muscle [98,133,135–137]. Moreover, desmin is required for proper distribution and morphology of mitochondria in muscle fibers, and both mitochondrial organization and morphology are altered during aging [97,138,139]. Finally, aging may impact desmin’s role in muscle adaptation due to altered desmin dynamics as a consequence of impaired desmin turnover [92].

### Age-related cytoskeletal changes and potential impact on nuclear mechanotransduction

Aging is associated with profound changes in non-sarcomeric cytoskeletal composition and organization in many cell types, including skeletal muscle [31,140]. There is some evidence to suggest that during murine aging (2, 14, and 23-month mice), the MT network undergoes changes that affect its structure, including a less dense network with fewer longitudinal bundles [31]. Nevertheless, a detailed characterization of the organization and PTM landscape is still lacking. Much of what we know about MTs alterations are from disease models.

Critically, these changes in the non-sarcomeric cytoskeleton could alter the mechanical load on the nucleus, which is directly connected to the cytoskeleton via the LINC complex, thereby contributing to the elongated nuclear morphology often observed in aged muscle fibers [31–33]. Thus, one possibility is that cytoskeletal remodeling underlies these age-dependent alterations, akin to work in cardiac myocytes showing acute disruption in the cytoskeletal network, specifically MTs, which impact nuclear architecture and mechanotransduction [100,101,141]. Similarly, p38 mitogen-activated protein kinase (p38/MAPK) signaling regulates cytoskeletal remodeling and differentiation in skeletal muscle [142] and is moderately dysregulated in a muscle-specific manner during aging [143], and could therefore influence myonuclear mechanotransduction indirectly through effects on microtubule organization and nuclear – cytoskeletal coupling. Although the cytoskeleton has traditionally been studied in the context of sarcomeric function, its nuclear interface is increasingly recognized as both a structural barrier and a mechanotransduction hub [144], mediating force transmission while also regulating access to signaling molecules and chromatin architecture [20,83]. Therefore, disruptions in cytoskeletal architecture with aging not only impair muscle contraction but also could compromise the transmission of mechanical signals to the nucleus, impairing gene expression and chromatin remodeling [20,31,83]. Nevertheless, a detailed characterization of how aging affects physical connections between the cytoskeleton and myonucleus is still lacking. To understand how aging may disrupt these connections, the next section examines how this stress is transmitted through the perinuclear cytoskeleton to impact nuclear architecture and function.

## Perinuclear cytoskeleton

### Composition and function of the perinuclear cytoskeleton in muscle fibers

The perinuclear cytoskeleton, or ‘nuclear cage,’ refers to the structural cytoskeletal matrix surrounding the nuclear periphery in muscle fibers [22,31,100]. This nuclear cage acts as a physical buffer, distributing forces around the nucleus via linkages between the nuclear envelope, lamina, and cytoskeletal elements (including MTs and desmin in striated muscle). The MT organization shifts from being a radial aster array from the centrosome in myoblasts toward a grid-like network seen in mature muscle fibers, with the nucleus acting as an organizing center [21,115,117,118]. The MT component of the nuclear cage exerts compressive forces that help maintain nuclear morphology and protect nuclei from mechanical stress [20,100,108]. Desmin indirectly connects to Nesprin 3 via the linker protein plectin-1 [145], which has been proposed to provide a resting tensile force that opposes MT compression [100]. This may balance mechanical forces on the nucleus, preserving nuclear shape and size and maintaining the integrity of the nuclear lamina and chromatin organization [145].

Experiments linking the loss of the MT cage to functional defects in adult muscle typically focus on genetic or molecular disruptions of components that maintain the MT nuclear cage and consequent muscle impairments [100,146,147]. A key study that directly visualized the MT nuclear cage in Drosophila skeletal muscle fibers showed that this is dependent on the LINC complex component Msp300 (a KASH (Klarsicht/ANC-1/Syne Homology) domain protein). Msp300 depletion resulted in altered nuclear positioning, nuclear envelope deformation, and impaired force transmission in muscle fibers. These structural defects were associated with reduced muscle contractility and dysfunctional mechanotransduction, linking MT cage loss directly to muscle functional decline. This is consistent with recent work showing that in mouse cardiomyocytes, disrupting the LINC complex eliminates the perinuclear MT cage [148], suggesting that a physical connection to the nucleus is required for MT cage formation.

### Age-related nuclear cage remodeling and potential impact on nuclear mechanotransduction

Few studies have examined how aging affects the nuclear cage in muscle fibers. Early work found that elongated myonuclei were associated with dense, ‘tail-like’ MT bundles that extended from the polar regions of nuclei, whereas nuclei surrounded by an evenly distributed MT cage tend to be rounder [20,31]. With aging, the structural integrity of the MT-desmin nuclear cage may be compromised, disrupting the force balance on myonuclei [21]. Studies have shown altered spacing between myonuclei [31,149], nuclear envelope invaginations [83], and perinuclear cytoskeletal remodeling in aged muscle fibers [31]. This may weaken the mechanical support that the nuclear cage provides, making myonuclei more susceptible to abnormal deformations resulting from the high mechanical demand exerted by contracting muscle fibers. Compromise of the nuclear cage can lead to nuclear shape changes [3,31–33,83], increased nuclear envelope infolding [83], elevated DNA damage [150], changes to gene expression [52], and disrupted nuclear envelope integrity [3,83]. More specifically, similar to *mdx* mice [15], we hypothesize that age-related alterations in perinuclear MT organization, which may include an accumulation of stable, detyrosinated microtubules or changes in desmin distribution, could alter mechanical feedback from the cytoskeleton to the nucleus, potentially compromising nuclear integrity. These alterations may reduce the efficiency of mechanosensitive pathways that regulate muscle maintenance and regeneration. Central to this mechanical coupling is the LINC complex, which bridges the cytoskeleton and nuclear lamina. The next section explores how age-related remodeling of LINC complex components might further impair nuclear mechanotransduction.

## LINC complex

### Structure and function of the LINC complex

The LINC complex comprises a group of proteins forming a conserved molecular bridge that spans through the nuclear envelope (NE) and anchors to the nucleoskeleton [151–153]. It forms a direct physical connection between the nucleus and the cytoskeleton, facilitating mechanotransduction and nuclear positioning in muscle fibers [39,154,155]. The NE comprises a double membrane bilayer composed of the inner (INM) and outer (ONM) nuclear membrane separated by roughly 50 nm perinuclear space (PNS). This NE acts as a physical barrier, separating the cytoplasm from the nucleoplasm [156]. Central to the LINC complex are two classes of transmembrane proteins: SUN (Sad1p and UNC-84) proteins embedded within the INM, and Nesprin (NE spectrin-repeat proteins) proteins localized primarily to the ONM [37,39,152], with some early reports suggesting smaller isoforms can be found in the INM interacting with the nuclear lamina and emerin [157,158], although whether this occurs in skeletal muscle is still debated [159]. The SUN domain located at the C-terminal end of SUN proteins physically interacts with the KASH peptide, a conserved protein motif located at the C-terminus of Nesprins [160], in the PNS to form a SUN-KASH interaction that couples the nucleus to the cytoskeleton physically and mechanically [39,152,153]. To date, five SUN genes (*SUN1-SUN5*) and four Nesprin genes (*SYNE1-SYNE4*) have been identified in vertebrates, encoding distinct SUN and Nesprin proteins [39]. Of those, *SUN1*, *SUN2, SYNE1*, and *SYNE2* genes are the most widely studied and expressed in skeletal muscle [161]. Alternative splicing and transcription start sites of Nesprins −1 and −2 may generate numerous potential isoforms, often in a tissue-specific manner [161], with the so-called giant isoforms interacting with actin via their calponin homology (CH) domain and/or microtubules via a LEWD motif-kinesin-1 interaction [162]. In addition, smaller isoforms can interact with microtubules via various interacting partners, including AKAP450 (aka AKAP9) [163,164]. Nesprin-3 interacts with intermediate filaments via the cytoskeletal linker protein plectin [165,166]. In addition, SUN2-containing LINC complexes have been reported to be more involved in actin–nucleus interactions, whereas SUN1-containing LINC complexes are more involved in microtubule–nucleus interactions [167,168], which has recently been shown to arise from specificity within their SUN domains, rather than their coiled-coil or nucleoplasmic domains [169]. This diversity enables the possible formation of a broad spectrum of LINC complexes tailored to specific cytoskeletal links and mechanobiological functions in muscle fibers [154,170], while age-related changes in the expression and/or localization of LINC complex proteins could alter specific nucleo-cytoskeletal connections in skeletal muscle fibers.

### The role of the LINC complex in skeletal muscle

Much of the role of the LINC complex in skeletal muscle has focused on its vital role in myonuclear positioning and anchorage during muscle development [94,154,155,163,171]. For example, Nesprin-1 is required for myonuclear anchoring in skeletal muscle [154,155]. Ablation of all Nesprin-1 isoforms in cardiomyocytes combined with Nesprin-2 global KO (gKO) led to a cardiomyopathy phenotype that was accompanied by changes to nuclear morphology, chromatin decondensation and defective mechanotransduction [172]. Moreover, SUN1 and SUN2 double knockout mouse models demonstrate disrupted nuclear positioning in skeletal muscle fibers [173]. In *Drosophila* larval muscles, disruption of the LINC complex affects the mechanical properties of myonuclei [174], in part by limiting chromatin repression [175]. Nevertheless, the role of the LINC complex in adult mammalian muscle fibers is less well understood. For example, disruption of the LINC complex in adult hearts using adeno-associated virus (AAV)-mediated transduction of a dominant-negative SUN1 construct, consisting of the luminal domain of Sun1 tagged at its NH2 terminus with HA [36], resulted in no overt cardiac phenotype or change in lifespan in wild-type mice [176]. Similarly, inducible expression of a dominant-negative KASH transgene in adult hearts resulted in a negligible effect on the transcriptome, with only four differentially expressed genes detected relative to animals with an intact LINC complex [177]. It should be noted that in these studies, molecular and functional changes were assessed relatively acutely following LINC complex disruption, and thus, defects may not have had time to manifest. Conversely, targeted ablation of Nesprin-1 in cardiomyocytes combined with Nesprin-2 gKO led to early onset cardiomyopathy [172]. It remains to be determined whether the method and timing of LINC complex disruption (i.e. dominant-negative versus knockdown/depletion; developing vs. adult heart) lead to different functional outcomes.

### Aging and the LINC complex

The majority of studies investigating how aging affects the composition and function of LINC complexes have used non-muscle cells. However, skeletal muscle fibers are multinucleated with distinct mechanical and structural forces, which may limit the interpretation of findings in cell culture. To bridge this gap in understanding, we recently described the relationship between aging, exercise training and myonuclear structure and mechanics in human and mouse muscle [3]. We observed elongated myonuclei that were more deformable upon sarcomeric stretching and had reduced lamin A deposition in older untrained individuals compared with age-matched, trained individuals. Interestingly, myonuclei were elongated in young, untrained individuals compared to trained counterparts, suggesting exercise-dependent, age-independent alterations in myonuclear parameters. In support of this, exercise training in mice led to increased nuclear stiffness, and increased levels of lamin A and SUN2. Interestingly, other LINC components such as SUN1, Nesprin-1α2, and Nesprin-2α1 were unchanged [3], suggesting a potential differential response of LINC complex proteins to exercise. It is tempting to speculate that increased SUN2 and Lamin A may contribute to myonuclear shape changes, highlighting a mechanistic link between LINC complex remodeling, nuclear architecture, and positioning during muscle aging.

## Nuclear lamina

Nuclear lamina is a dense fibrillar network located beneath the INM, composed of type V intermediate filaments proteins called Lamins A, B1, B2, and C [21,41]. The nuclear lamina plays a crucial role as a ‘shock absorber’ for the nucleus during repetitive contractions [21,178], as well as providing nuclear stiffness [179,180], maintaining nuclear integrity (3,29,180,182), and regulating gene expression [179,181]. The importance of the nuclear lamina, particularly Lamins A and C, in skeletal muscle is evident from mutations in the *LMNA* gene, which give rise to a class of diseases (i.e. laminopathies) that largely affect striated muscles (cardiac and skeletal) [182]. Muscular laminopathies display pronounced muscle weakness, abnormal nuclear shapes, nuclear invaginations, and defective mechanotransduction. A-type lamins (Lamin A/C) and B-type lamins have distinct functions in nuclear mechanics and gene regulation [183]. Recent work in mouse embryonic fibroblasts (MEFs) demonstrated that A- and B-type lamin isoforms play distinct roles in nucleocytoskeletal connectivity, where A-type lamins engage with F-actin through LINC complexes to modulate cortical stiffness and contractility, while B-type lamins predominantly interact with vimentin intermediate filaments to regulate cytoplasmic stiffness [44]. Thus, the nuclear lamina may also act as an ‘inside-out’ regulator of cell mechanotransduction, serving to influence the adaptive response of muscle cells to mechanical cues.

### A-type lamins

Lamin A/C, encoded by the *LMNA* gene, is highly expressed in mechanically active tissues, including skeletal muscle [179]. Lamins A and C arise from alternative splicing of the *LMNA* transcript [184,185]. After synthesis, Lamin A undergoes PTMs including farnesylation at its C-terminal CaaX motif, but unlike B-type lamins, mature Lamin A undergoes a cleavage step where the farnesyl group and additional 15 amino acids at its C terminus are removed [185]. Lamin C, which lacks the CaaX motif, is not farnesylated [184,185]. Lamin A and Lamin C form filament networks adjacent to the INM but are organized in distinct, partly overlapping networks separate from B-type lamins [186]. The farnesylation of Lamin A and B-type lamins helps target them to the nuclear envelope through hydrophobic interactions, which is key for nuclear lamin assembly [184]. During nuclear envelope formation, Lamin A interacts with proteins like barrier-to-autointegration factor (BAF) and factors such as Emerin, ensuring even Lamin A distribution [187]. Lamin A/C expression levels scale with tissue stiffness, determining the density and organization of the nuclear lamina meshwork and thereby regulating nuclear mechanical properties [179]. A key mechanism by which Lamin A/C influences transcriptional regulation is via lamina-associated domains (LADs), genomic regions that interact directly with the nuclear lamina and are generally associated with transcriptional silencing [188]. Mechanical deformation of the lamina, as occurs during muscle contraction [189], may serve to displace LADs from the lamina, altering chromatin accessibility and enabling the expression of mechanosensitive genes. Beyond the nuclear periphery, chromatin is also regionally segregated at the nucleolar surface through Nucleolus-Associated Domains (NADs), genomic regions enriched in repressive chromatin marks (e.g., H3K9me2/3) that associate with the nucleolar periphery in a manner analogous to LADs at the nuclear lamina [190]. Many loci stochastically associate with either compartment, suggesting the nucleolus and nuclear lamina cooperate to separate large portions of the genome into transcriptionally silent domains [191,192]. In skeletal muscle, the nucleolus undergoes marked structural reorganization during myogenic differentiation [193], with myotubes harboring fewer, larger nucleoli reflecting the shift toward sustained ribosome production for contractile protein synthesis. With aging, we have shown that rDNA transcriptional activity declines, changes that may contribute to the transcriptional dysregulation observed in aged myonuclei [194].

Together, the cytoskeleton-LINC-lamina-chromatin axis constitutes a continuous mechanotransductive pathway in which contractile forces could deform the nuclear lamina, reorganize chromatin architecture through LAD and/or NAD displacement and emerin-mediated transcriptional regulation, and ultimately modulate the gene-expression programs necessary for muscle maintenance and adaptation.

### B-type lamins

B-type lamins are composed of Lamin B1 and Lamin B2, respectively encoded by the genes *LMNB1* and *LMNB2*, together forming a major structural element of the nuclear lamina [83,195,196]. B-type lamins are required for normal tissue development, in particular of the central nervous system (CNS) [197]. While it is not typically thought that B-type lamins are key contributors to the mechanical properties of the nucleus [183], recent work has shown that Lamin B1 works cooperatively with the INM protein LAP2β the help organize the nuclear lamina and protect the nucleus from mechanical stress [198] and Lamins B1 and B2 play distinct roles in neurons to maintain nuclear integrity [197]. In addition, B-type lamins contribute to nuclear stiffness, but only in the absence of A-type lamins [199].

### The importance of the nuclear lamina in skeletal muscle health

Global Lamin A/C knockout strains have been invaluable for gaining mechanistic insights into the role of the nuclear lamina in muscle [30,200]. *Lmna* KO mice have a severe whole-body phenotype, including reduced growth, muscular dystrophy, dilated cardiomyopathy, peripheral neuropathy, and lipodystrophy, making it challenging to uncouple primary from secondary effects in the muscle. The generation of *Lmna* floxed mice [201] has enabled direct interrogation of the importance of the nuclear lamina in skeletal muscle [181,202]. Muscle-specific deletion of *Lmna* leads to NMJ degeneration, which precedes muscle fiber defects, including reduction in muscle fiber cross-sectional area and central nucleation [202]. The degeneration of the NMJ is consistent with results from another study, which reported a decrease in absolute muscle force production with *Lmna* deletion, albeit some of the reduced force is likely explained by the reported decrease in muscle size [181]. In addition, the study by Xiong and colleagues reported that loss of Lamin A/C in skeletal muscle led to muscle aging-like deficits, reduced muscle mass, kyphosis, and increased cellular senescence markers such as senescence-associated-β-galactosidase (SA-β-gal), p16^Ink4a^, and p53 [181]. Notably, Lamin A/C-deficient muscle fibers secreted higher IL-6, promoting osteoclast differentiation and bone resorption, directly linking muscle aging to osteoporosis. This study establishes a mechanistic connection between loss of lamin A/C in muscle, cellular senescence, chronic inflammation, and bone deterioration in aging. Of note, the aforementioned studies all use a constitutive skeletal muscle-specific Cre recombinase mouse strain [203]; thus, some of the defects may still arise during muscle development. Future work using an inducible-skeletal muscle-specific Cre strain [204] will help to elucidate how loss of lamin expression in adult tissue affects muscle health and function. Recent studies using this inducible-depletion of *Lmna* in adult hearts showed a rapid decline of cardiac function within ~3 weeks [176,177,205], consistent with the recently reported half-life of Lamin A/C in the heart [206].

### The nuclear lamina in aged skeletal muscle

Evidence indicates that the nuclear lamina may undergo molecular and structural changes with aging [3,21]. One possibility for the abnormal nuclear morphology with aging would be the accumulation of prelamin A, a precursor of mature Lamin A, which has been shown to accumulate in aged cells, including smooth muscle cells [207]. To this end, forced accumulation of prelamin A in skeletal muscle fibers through deletion of *Zmpste24*, the enzyme responsible for producing mature lamin A protein, leads to interference with the nuclear lamina structure, causing an increase of nuclear stiffness, DNA damage, and impaired contraction [184,185]. Nevertheless, *ZMPSTE24* expression does not change with age in skeletal and cardiac cells [184].

Aging may lead to changes in the expression of nuclear lamina proteins. However, studies in aged skeletal muscle generally show mixed results, with some indicating little change in protein levels while others reveal functional or interaction modifications [83,181,202]. In humans, *LMNA* expression has been reported to increase with age, but this did not coincide with an increase in LMNA protein levels [178]. In mice, Lamin A/C protein levels appear to decline between 3- and 24-months of age and have been reported to coincide with NMJ fragmentation and denervation, further worsening muscle weakness and contractile defects [202]. This suggests that muscle aging may involve Lamin A/C alterations beyond simple expression changes, such as post-translational modifications, as both unchanged and decreased *LMNA* expression are associated with age-specific nuclear alterations [178,181].

To regulate nuclear stability, Lamin A/C must be properly assembled at the nuclear periphery. If nuclear lamina assembly or organization is altered with aging, this could lead to altered nuclear morphology and mechanotransduction. Regular exercise leads to greater myonuclear sphericity, increased expression, and deposition of Lamin A and restores nuclear stiffness, suggesting that exercise may counteract some age-related changes in nuclear lamina structure and function [3]. However, such exercise-driven remodeling is largely independent of age, supporting the concept that mechano-adaptation via lamina preservation is key to healthy muscle aging [3].

Lamin B1 is often used as a marker for cellular senescence during aging. Recent studies consistently show that lamin B1 protein levels decline in aged skeletal muscle cells, with the reduction most pronounced in synaptic nuclei beneath the neuromuscular junction, as observed in aged murine muscle [83,195,196,208]. Notably, this age-related depletion appears to be specific for Lamin B1 as Lamin B2 protein remains unchanged [83]. In parallel with Lamin B1 loss, decreased levels of nuclear pore complex (NPC) protein Nup107 and increased levels of Nup93 have been observed in aged skeletal muscle [83]. These changes are thought to be associated with altered nuclear morphology and enhanced nuclear permeability, or ‘leakiness,’ indicative of compromised nuclear envelope [83,196]. The functional effect of reduced Lamin B expression in aged muscle requires further interrogation.

### Integrative perspective and future directions

The nucleus represents the ultimate mechanosensitive hub in skeletal muscle, integrating signals from the ECM via integrins, the cytoskeleton, and converging on the nucleus. Aging perturbs the continuum at each level of this chain (Table 1), but the consequences are most noticeable at the nucleus, where structural instability [3,21,83], altered mechanotransduction [3,16], and transcriptional dysregulation [16] that may collectively contribute to muscle function decline [16,22]. Whether these nuclear changes represent adaptive remodeling or secondary consequences of altered mechanical loading remains an open question. To move beyond association toward causality, future studies will need to employ muscle-specific models that target individual components of the nuclear mechanotransduction pathway or disrupt the LINC complex globally, alongside aging protocols and assessment of muscle quality and function. In addition, determining which alterations in the nuclear mechanotransduction cascade are conserved across species will help inform where to focus for potential intervention development.

In healthy fibers, ECM-integrin-cytoskeleton linkages distribute mechanical loads evenly across myonuclei, allowing them to deform without losing structural integrity [209]. With aging, fibrotic ECM stiffening distorts load transmission and may activate maladaptive mechanosensitive pathways such as YAP/TAZ or myocardin-related transcription factor A (MRTF-A) in SCs and FAPs [54,82]. However, whether this occurs in multinucleated fibers has yet to be determined. Targeted approaches, like single-fiber isolation and culture on substrates of varying stiffness as well as spatial multiomics (epigenomics, transcriptomics, and proteomics) approaches, could help clarify cell type-specific contributions.

Age-related remodeling of the non-sarcomeric cytoskeleton elements (MTs and desmin) further disrupts force transmission [14,15,31], stiffens the cytoskeleton, compromises myofibrillar organization [133,134], and increases nuclear load via the LINC complex. Loss of MT lattice integrity, together with increased stiffness, may push fibers beyond an optimal stiffness and impair mechanosensitivity [49]. However, direct evidence in aged skeletal muscles is limited, underscoring the need for careful, longitudinal profiling of age-dependent MT composition and modifications. Studies in cardiac muscle demonstrate that such MT remodeling alters nuclear shape and mechanotransduction [100,101,141]. PTM-driven MT stiffening stabilizes the MT cage and increases compressive forces, while desmin misalignment reduces tensile support, leading to nuclear deformation, envelope invaginations, and impaired mechanosensing [100]. Paradoxically, acute disruption of the MT cage in both cardiac [100] and skeletal muscle [210] produces elongated nuclei similar to those seen in aged fibers. These findings suggest that MT alterations may differ between the perinuclear and cytoplasmic regions. In aging, the cytoplasmic region likely shows, similar to *mdx* mice [15], increased MT-PTM levels leading to cytoskeletal stiffening. Conversely, the perinuclear region may exhibit reduced PTM levels contributing to both age-dependent cytoskeletal stiffening and myonuclear elongation. To clarify these compartment-specific changes and to separate cytoplasmic and perinuclear contributions requires high-resolution imaging and targeted perturbations enabling detailed analysis of single muscle fiber regions.

Most studies focusing on aging-related LINC complex changes in muscle often occur alongside alterations in the nuclear lamina, prompting further exacerbation of nuclear fragility. Specifically, inactive aging in muscle is associated with elongated myonuclei, reduced lamin A deposition, and decreased nuclear stiffness compared to age-matched exercise-trained individuals [3]. In contrast, SUN1 and Nesprin isoforms remain largely stable with age, though SUN1 loss in development causes nuclear mislocalization [173], emphasizing the importance of preserved nuclear membrane architecture. Declines in the integrity of lamin A [202] and B1 [83,196] contribute to nuclear fragility [202], reduced stiffness (3,202), and leakage across the nuclear envelope [83,196], promoting DNA damage, chromatin disorganization, and cellular senescence that ultimately leads to functional decline, including muscle weakness. Exercise, conversely, enhances nuclear sphericity as well as lamin A and SUN2 expression [3], suggesting that mechanical activity preserves nuclear envelope stability and function across ages. Further work is needed to define whether aging affects the composition or the distribution of LINC complex components in muscle fibers.

Taken together, these findings position aged myonuclei as recipients of impaired force transmission and potentially active participants in contributing to muscle dysfunction. To validate the active participation, functional measurements of myonuclei are necessary by measuring the temporal nuclear morphology changes during contraction. Direct acute experimental disruption of the microtubule cage in adult skeletal muscle fibers with paired contractile force measurements appears to be lacking. Of interest would be using newly developed optogenetic systems [211] to achieve acute MT cage disruption coupled with real-time contractility [212] to define the role of MTs on contraction-induced nuclear deformation. However, obtaining myonuclear shape during muscle contraction remains challenging, as the contractile and relaxation speeds are much faster compared to those in cardiac myocytes, in which recent studies have tracked nuclear deformation during contraction [141,213,214]. Existing experiments largely focus on chronic or genetic disruptions of cytoskeletal components or broader muscle damage models that impact multiple systems, including the MT cage. Thus, direct experiments acutely disrupting the MT cage in adult myofibers and measuring contractile force have not yet been reported.

Future work looking at the effect of aging on structures involved in nuclear mechanotransduction in human subjects should make use of longitudinal studies, as most evidence relies on interpreting single-time point, cross-sectional data from different individuals, which undoubtedly introduces various confounding factors. Also of interest would be dissecting the mechanisms by which physical activity influences nuclear shape [3], and whether human muscle nuclei revert to an elongated shape following a period of detraining, as has been shown in mouse models [215]. It is intriguing to speculate that altered nuclear mechanotransduction contributes to the recently described differential transcriptome and epigenome response to an acute mechanical stimulus in aged murine myonuclei [216]. Whether this is also the case in humans remains to be evaluated.

## Conclusion

Collectively, evidence indicates that the myonucleus might be a critical nexus in muscle aging, where mechanical inputs from the ECM and cytoskeleton converge and are transduced into transcriptional outcomes. Aging may destabilize this nuclear hub at multiple levels (the perinuclear cytoskeleton, LINC complex, and lamina), leading to impaired force buffering, nuclear fragility, and maladaptive gene expression. Critically, these nuclear changes are unlikely to be purely passive consequences of upstream cytoskeletal dysfunction; through inside-out signaling, a destabilized nuclear lamina and altered chromatin state may in turn compromise cytoskeletal organization and mechanosensitivity, positioning the aged myonucleus as an active contributor to the broader cycle of muscle deterioration. By focusing research on the nucleus as the central integrator of age-associated mechanobiology, we might better understand how structural changes lead to functional decline. Future studies integrating biophysical assays, imaging, and multiomics approaches will be essential to mechanistically link mechanical defects to nuclear and transcriptional dysfunction, potentially shaping strategies to preserve muscle function with age.

## Data Availability

Data sharing does not apply to this article as no new data were created or analyzed in this study.
